# Prevalence of different parasomnias in the general Norwegian population, and their association with insomnia, anxiety, and depression. A cross-sectional web-panel survey

**DOI:** 10.3389/frsle.2026.1806980

**Published:** 2026-04-23

**Authors:** Erlend Haarr Drugli, Oskar Emil Lehmann, Ståle Pallesen, Ingvild West Saxvig, Siri Waage, Bjørn Bjorvatn

**Affiliations:** 1 Department of Global Public Health and Primary Care, University of Bergen, Bergen, Norway; 2 Norwegian Competence Center for Sleep Disorders, Haukeland University Hospital, Bergen, Norway; 3 Department of Psychosocial Science, University of Bergen, Bergen, Norway

**Keywords:** anxiety, circadian preference, depression, epidemiology, insomnia, Norway, parasomnia

## Abstract

**Objective:**

To estimate the prevalence of various parasomnias in the general Norwegian adult population and explore their associations with insomnia, anxiety, and depression.

**Methods:**

A cross-sectional online survey was conducted in September 2024 among 1002 adults (50.7% male, mean age 50.3), drawn from a large population-based sample from a national web panel. Participants reported lifetime and past 3-month (current) prevalence of eleven different parasomnias, including both NREM- and REM-related subtypes. Validated instruments assessed insomnia (Bergen Insomnia Scale), anxiety and depression (Patient Health Questionnaire-4). Associations were analyzed using chi-square tests and logistic regressions, adjusting for age, sex, educational level, and circadian preference. Response rate was 19.8%.

**Results:**

Lifetime prevalence of the parasomnias ranged from 2.3% (sleep-related eating disorder) to 47.2% (nightmares), while current prevalence ranged from 1.0% (injured somebody else during sleep) to 33.1% (nightmares). Insomnia, anxiety, and depression were associated with most of the parasomnias [e.g., nightmares-anxiety (OR 1.58; CI 1.42–1.74)]. Parasomnias were more common in younger participants, while sex differences were few.

**Conclusion:**

Many of the parasomnias were commonly reported and strongly associated with insomnia and mental health symptoms. These findings underscore the need for increased clinical awareness and further research on parasomnias.

## 1 Introduction

Parasomnias are undesirable physical events or experiences that occur during entry into sleep, within sleep, or during arousal from sleep (American Psychiatric Association, 2013). They are divided into three groups depending on whether they occur during non-rapid eye movement (NREM) sleep, rapid eye movement (REM) sleep, or during any sleep stage. NREM-related parasomnias arise due to incomplete arousals from deep sleep (American Psychiatric Association, 2013). The International Classification of Sleep Disorders, version 3 (ICSD-3) includes confusional arousals, sleepwalking, sleep terrors, and sleep-related eating disorder within this category, while violent behavior during sleep (both toward others and self) is recognized as an associated feature (American Academy of Sleep Medicine, 2014). Sleep-related sexual behavior or sexsomnia is regarded as a clinical subtype of confusional arousals (American Academy of Sleep Medicine, 2014). REM-related parasomnias include REM sleep behavior disorder (RBD), recurrent isolated sleep paralysis, and nightmare disorder (American Academy of Sleep Medicine, 2014). The third category comprises parasomnias not related to any specific sleep stage and includes exploding head syndrome, sleep-related hallucinations, and sleep enuresis (American Academy of Sleep Medicine, 2014). For the purpose of this study, all parasomnia events, along with their associated features and subtypes, are defined as parasomnias.

According to a Norwegian epidemiological study from 2010, the lifetime prevalence of different parasomnias in the general adult population ranges from 3.8% (injured somebody else during sleep) to 66.2% (nightmares), whereas current prevalence (last 3 months) ranges from 0.4% (injured somebody else during sleep) to 19.4% (nightmares), with few sex differences (Bjorvatn et al., 2010). That study did not explore whether there were age differences in the prevalence of parasomnias. Nevertheless, several studies show a decreasing tendency of most parasomnias with increasing age. This pertains particularly to the NREM-parasomnias and nightmares (Nielsen et al., 2006; Ohayon et al., 1999; Ohayon and Schenck, 2010; Takaesu et al., 2016; Trajanovic et al., 2007). Conversely, RBD is associated with neurodegenerative diseases and shows accordingly a higher prevalence with increasing age (St Louis et al., 2017). To our knowledge, no association between age and sleep paralysis or exploding head syndrome has been reported in previous studies (Denis et al., 2019; Sharpless and Barber, 2011).

Although epidemiological evidence on the relationship between circadian preference and parasomnias is scarce, emerging research suggests that circadian preference is related to parasomnia-like experiences. Various chronobiological mechanisms have been proposed to play an important role in NREM-parasomnia (Mainieri et al., 2021). Evening chronotype is associated with greater nightmare distress (Choo et al., 2023). In contrast, a study investigating associations between chronotype and exploding head syndrome found no significant relationship (Kirwan and Fortune, 2021). These findings suggest that circadian preference is relevant when investigating the occurrence of parasomnias.

In terms of associations with mental health, a cross-sectional study among 1,296 university students in Saudi Arabia found a significant association with having a parasomnia and insomnia, anxiety and depression (Alshahrani et al., 2023). This study did not specify type of parasomnia, warranting further research. In addition, a retrospective study among patients at US sleep centers reported an association between psychiatric illness and self-reported parasomnias, as well as insomnia to parasomnias (Hanif et al., 2024). Among the parasomnias explored were sleepwalking, sleep-related eating disorder and sleep paralysis (Hanif et al., 2024). The association between insomnia and parasomnias could be partly explained by the effects of sleep deprivation in already vulnerable patients, as shown in a sleepwalking study (Pressman, 2007). In that study, insomnia is understood as a priming factor, with other predisposing factors, such as genetic vulnerability, as well as precipitating factors, such as internal or external stimuli, likely to play important roles. The sleep disturbing nature of parasomnias could also have an effect on sleep duration and sleep stage transitions, suggesting a bidirectional relationship between insomnia and parasomnias. Another mediating factor is the use of sleep medications, such as zolpidem, which has been shown to trigger parasomnias (Ho et al., 2020; Mittal et al., 2021; Stallman et al., 2018). More research investigating the possible association between different types of parasomnias with insomnia, anxiety, and depression, is warranted, especially among the general population. In line with this, several scholars have addressed the need for additional studies exploring parasomnias and their associated factors (Bjorvatn et al., 2010; Alshahrani et al., 2023; Hanif et al., 2024; Gao et al., 2023).

Against this backdrop, we aimed to estimate the lifetime prevalence of different parasomnias in the general Norwegian adult population. We also aimed to investigate whether current parasomnias, defined as reporting occurrence at least once during the last 3 months, were associated with demographic factors like age and sex, in addition to circadian preference. Additionally, we aimed to explore to which degree current parasomnias were related to symptoms of insomnia, anxiety, and depression.

## 2 Methods

### 2.1 . Procedure

Data were collected during September 2024 by the polling agency Opinion and their subcontractor Norstat, using an online questionnaire. The sample was recruited from a web panel comprising 100,000 individuals who have consented to participate in research and various polls. Demographic groups with poor response rates were oversampled in order to obtain a sample representative for the population in terms of age, sex, and geography. Before responding to the survey, participants were informed that the survey included questions about different sleep phenomena and sleep-related habits, as required by the ethics committee. They were briefed that they could abort at any time during completion of the survey without any consequences. The Regional Committee for Medical and Health Research Ethics South-East Norway approved this study (no. 763265).

### 2.2 Participants

Out of 5,055 invited individuals, 1,002 completed the survey, resulting in a response rate of 19.8%. The planned sample size was 1,000, in accordance with previous articles, assumed to reflect representative data from the population (Bjorvatn et al., 2010). The crude sample consisted of 50.7% males, with a mean age of 50.3 years (SD 17.5, range 18–88 years).

### 2.3 Material

Data regarding sociodemographic parameters were provided by the polling agency: Sex (male; female), age, and educational level (primary school; high school; post-secondary education of 1–2 years; university/college up to 3 years; university/college 4 or more years; other). Age was later categorized into the following groups: 18–35 years, 36–50 years, 51–65 years, 66 years and older. Educational level was re-categorized into lower education (first two response categories), intermediate education (third response category), and higher education (last two response categories). In all, 7 participants responded “other” educational level and were excluded from these analyses. Circadian preference was assessed with the item “Are you a morning- or an evening person?” The participants responded on a five-point scale: “I am very alert and active in the morning, and sleepy early in the evening (definitely morning preference)”, “I am rather alert in the morning and rather sleepy in the evening (morning preference)”, “Neither morning nor evening person (intermediate preference)”, “I am rather alert in the evening and rather sleepy in the morning (evening preference)”, and “I am very alert and active during the evening, and sleepy during the morning (definitely evening preference)” (Saxvig et al., 2023). This variable was grouped into three categories: morning preference for the first two response categories, intermediate preference for the third response category, and evening preference for the last two.

The participants were asked about lifetime prevalence of eleven different parasomnias. The parasomnias explored in the survey were as follows: confusional arousals, sleepwalking, sleep terrors, sleep-related eating disorder, violence toward others, self-harm, sexsomnia, dream enactment, sleep paralysis, nightmares and exploding head syndrome (Table 1). The response alternatives were “yes” and “no” for each item, except for the one regarding sexsomnia, for which the participants could tick off “Would like not to answer”. In the further analysis of the latter variable, the participants who were unwilling to answer were excluded, totaling 34 participants. If endorsing any of the parasomnias, the participants were asked about the frequency of the specific parasomnias during the last 3 months on a six-point scale: “never”, “less than once per month”, “less than once per week”, “1–2 times per week”, “3–5 times per week”, and “daily/almost daily”. We defined current prevalence as having experienced the parasomnia in question at least once during the last 3 months. The parasomnia questions were phrased similarly to the ones in the article from 2010 (Bjorvatn et al., 2010), with a few exceptions. The previous study from 2010 only asked about the general experience of nightmares (Bjorvatn et al., 2010). In the present study, we added the requirement that the nightmares should be “repeated and terrible”, in accordance with ICSD-3 (American Academy of Sleep Medicine, 2014). The present survey also included questions regarding sleep paralysis and exploding head syndrome, in contrast to the previous article.

**Table 1:** Parasomnia questionnaire.

1. Have you ever experienced or been told that you have woken up at night in a confusional state without remembering the event the next day?
2. Have you ever experienced or been told that you have been sleepwalking?
3. Have you ever experienced or been told that you have woken up at night in terror without remembering the event the next day?
4. Have you ever experienced or been told that you have eaten food in your sleep?
5. Have you ever experienced or been told that you have injured somebody else during sleep?
6. Have you ever experienced or been told that you have injured yourself during sleep?
7. Have you ever experienced or been told that you have performed sexual acts in your sleep?
8. Have you ever experienced or been told that you have been acting out of a dream in your sleep?
9. Have you ever experienced that you have recurrent paralysis in your body, arms and legs at sleep onset or upon waking from sleep?
10. Have you ever experienced repeated and terrible nightmares in your sleep?
11. Have you ever experienced that you have been disturbed by a loud noise or explosion in your head either at sleep onset or waking during the night?

Insomnia symptoms were assessed with the validated Bergen Insomnia Scale (BIS), a questionnaire consisting of six items scored on an eight-point scale, representing the number of days per week (0–7) respondents experience the following: (1) spending more than 30 min falling asleep, (2) waking up more than 30 min during sleep, (3) waking up 30 min earlier than preferred, without falling asleep again, (4) not feeling adequately rested following sleep, (5) been so tired/sleepy that it affected work/school or your private life, (6) been dissatisfied with own sleep (Pallesen et al., 2008). The composite insomnia score ranges from 0–42, with a higher score indicating a higher symptom load. Chronic insomnia was defined as scoring at least 3 days per week on one or more of the first three items, as well as on one or more on the last two items, according to the criteria of the fifth edition of the Diagnostic and Statistical Manual of Mental Disorders (DSM-5) and the ICSD-3 (American Academy of Sleep Medicine, 2014). We assessed symptoms of depression and anxiety with the validated 4-item Patient Health Questionnaire-4 (PHQ-4). This brief self-report questionnaire consists of a 2-item depression scale (PHQ-2) and a 2-item anxiety scale (Generalized Anxiety Disorder assessment, GAD-2; Löwe et al., 2010). The composite scores on the subscales range from 0-6, with a higher score indicating a higher symptom load. Depression was defined as scoring 3 or more on the PHQ-2, and anxiety as scoring 3 or more on the GAD-2, in line with earlier studies (Kroenke et al., 2003, 2007).

### 2.4 Statistics

Data analyses were conducted using IBM SPSS Statistics 29 (SPSS Inc., Chicago, IL, USA). The analyses were weighted according to the population distribution by age, sex, and geography to account for potential discrepancies between the sample and the population. The weight factor ranged from 0.51 to 2.05 (mean 1.00, median 0.84, standard deviation 0.35). Relationships between current parasomnias (last 3 months) and insomnia, depression, and anxiety, as well as sex, age, and circadian preference, were explored using chi-square analyses. In addition, separate crude logistic regression analyses were conducted with the eleven parasomnias as the dependent variables, and sex, age, educational level, circadian preference, total insomnia score, total depression score, and total anxiety score as predictors. Lastly, separate adjusted logistic regression analyses (for sex, age, educational level, and circadian preference) were conducted with the total scores of insomnia, depression, and anxiety as predictors. The significance level was set to 0.05 in all analyses.

## 3 Results

Sociodemographic (sex, age, and educational level) and clinical characteristics (circadian preference, depression, anxiety, insomnia) are displayed in Table 2. The lifetime prevalence of the different parasomnias ranged from 2.3% (sleep-related eating disorder) to 47.2% (nightmares) (Table 3). Current prevalence was predictably lower and ranged from 1.0% (Injured somebody else during sleep) to 33.1% (nightmares). Occurrence at least once per week ranged from 0.0% (Injured yourself/somebody else during sleep) to 4.8% (nightmares). When looking at the total number of parasomnias reported, most participants had experienced either none (29.0%) or only one (26.2%) during their lifetime, while fewer reported two (18.4%) or three (12.4%). The prevalence declined steadily with an increasing number of parasomnias, and only 6.7% reported five or more parasomnias during their lifetime.

**Table 2:** Sociodemographic and clinical characteristics of the sample (*n* = 1,002).

Characteristic	Crude	Weighted
Sex		
Female	494 (49.3%)	500 (49.9%)
Male	508 (50.7%)	502 (50.1%)
Age		
18–35 years	248 (24.8%)	290 (28.9%)
36–50 years	246 (24.6%)	250 (24.9%)
51–65 years	268 (26.7%)	233 (23.2%)
66+ years	240 (24.0%)	230 (23.0%)
Educational level^a^		
Lower	232 (23.3%)	245 (24.6%)
Intermediate	182 (18.3%)	190 (19.1%)
Higher	581 (58.4%)	561 (56.3%)
Circadian preference		
Morning	345 (34.4%)	333 (33.3%)
Intermediate	262 (26.1%)	262 (26.2%)
Evening	395 (39.4%)	406 (40.5%)
Chronic insomnia (BIS)^b^		
No	638 (63.7%)	640 (63.9%)
Yes	364 (36.3%)	362 (36.1%)
Anxiety (GAD-2)^c^		
No	855 (85.3%)	854 (85.2%)
Yes	147 (14.7%)	148 (14.8%)
Depression (PHQ-2)^d^		
No	839 (83.7%)	833 (83.2%)
Yes	163 (16.3%)	169 (16.8%)

The data are represented both in crude and weighted form.

^a^*n* = 995.

^b^Bergen Insomnia Scale.

^c^Generalized Anxiety Disorder – 2.

^d^Patient Health Questionnaire – 2.

**Table 3:** Prevalence of different parasomnias (*n* = 1,002).

Type of parasomnia	Lifetime prevalence % (95%CI)	Current prevalence (at least once during the last 3 months) % (95%CI)	Current and occurring at least once a week % (95%CI)
Confusional arousals	19.3 (16.9–21.8)	11.8 (9.8–13.8)	0.9 (0.3–1.5)
Sleepwalking	18.5 (16.1–20.9)	3.9 (2.7–5.1)	0.3 (0.0–0.6)
Sleep terrors	11.6 (9.7–13.6)	8.8 (7.1–10.6)	0.5 (0.1–0.9)
Sleep-related eating disorder	2.3 (1.4–3.2)	1.3 (0.6–2.0)	0.2 (0.0–0.5)
Injured somebody else during sleep	3.0 (1.9–4.0)	1.0 (0.4–1.6)	0.0
Injured yourself during sleep	2.6 (1.6–3.6)	1.3 (0.6–2.0)	0.0
Sexsomnia^*^	10.9 (8.9–12.9)	6.3 (4.8–7.8)	0.7 (0.2–1.2)
Dream enactment	20.1 (17.7–22.6)	15.5 (13.2–17.7)	0.5 (0.1–1.0)
Sleep paralysis	18.8 (16.4–21.3)	15.0 (12.8–17.2)	2.9 (1.9–4.0)
Nightmares	47.2 (44.1–50.3)	33.1 (30.2–36.0)	4.8 (3.5–6.1)
Exploding head syndrome	12.9 (10.8–14.9)	10.8 (8.9–12.8)	1.5 (0.8–2.3)

^*^n = 968.

Lower CI limits were truncated at 0 due to the impossibility of negative prevalence.

All prevalence estimates are reported in percent, with 95% confidence intervals in parentheses.

Age was significantly associated with several of the current parasomnias. Confusional arousals, dream enactment, sleep paralysis, and nightmares all had a higher prevalence in the youngest age group (Table 4). Sleep terrors were most prevalent in the 51–65 year age group, while exploding head syndrome was most frequently reported by those below 50 years. Males reported current dream enactment more often than females (Table 4). Current nightmares were almost twice as prevalent in women as in men. Otherwise, no sex differences were detected. Participants with morning-oriented circadian preference reported the highest prevalence of sexsomnia, while evening types reported the highest prevalence of exploding head syndrome (Table 4). No other significant associations were found regarding circadian preference.

**Table 4A:** Current prevalence (at least once during the last 3 months) of different parasomnias in relation to sex and age group (*n* = 1,002).

Type of parasomnia	Sex			Age group				
	Male	Female	Chi-square	18–35 y	36–50 y	51–65 y	66 y+	Chi-square
Confusional arousals								
No	435 (86.7%)	448 (89.8%)	χ^2^ = 2.352	236 (81.4%)	229 (91.6%)	202 (87.1%)	217 (94.3%)	χ^2^**=** **24.419**
Yes	67 (13.3%)	51 (10.2%)	*p* = 0.125	54 (18.6%)	21 (8.4%)	30 (12.9%)	13 (5.7%)	***p*** **<** **0.001**
Sleepwalking								
No	478 (95.2%)	485 (97.2%)	χ^2^ = 2.673	273 (94.1%)	240 (96.0%)	226 (97.0%)	225 (97.8%)	χ^2^ = 5.328
Yes	24 (4.8%)	14 (2.8%)	*p* = 0.102	17 (5.9%)	10 (4.0%)	7 (3.0%)	5 (2.2%)	*p* = 0.149
Sleep terrors								
No	455 (90.6%)	458 (91.6%)	χ^2^ = 0.287	259 (89.3%)	241 (96.8%)	202 (87.1%)	211 (91.7%)	χ^2^ **=** **16.006**
Yes	47 (9.4%)	42 (8.4%)	*p* = 0.592	31 (10.7%)	8 (3.2%)	30 (12.9%)	19 (8.3%)	**p** **=** **0.001**
Sleep-related eating disorder								
No	496 (98.8%)	493 (98.6%)	χ^2^ = 0.082	289 (99.7%)	246 (98.8%)	227 (97.4%)	227 (98.7%)	χ^2^ = 5.042
Yes	6 (1.2%)	7 (1.4%)	*p* = 0.775	1 (0.3%)	3 (1.2%)	6 (2.6%)	3 (1.3%)	*p* = 0.169
Injured somebody else during sleep								
No	500 (99.4%)	493 (98.6%)	χ^2^ = 1.640	283 (97.6%)	249 (99.6%)	232 (99.6%)	228 (99.1%)	χ^2^ = 6.823
Yes	3 (0.6%)	7 (1.4%)	*p* = 0.200	7 (2.4%)	1 (0.4%)	1 (0.4%)	2 (0.9%)	*p* = 0.078
Injured yourself during sleep								
No	498 (99.0%)	491 (98.2%)	χ^2^ = 1.183	284 (97.9%)	244 (98.0%)	231 (99.1%)	230 (100.0%)	χ^2^ = 5.704
Yes	5 (1.0%)	9 (1.8%)	*p* = 0.277	6 (2.1%)	5 (2.0%)	2 (0.9%)	0 (0.0%)	*p* = 0.127
Sexsomnia^*^								
No	444 (92.5%)	463 (94.9%)	χ^2^ = 2.316	254 (93.7%)	226 (94.2%)	210 (91.7%)	218 (95.2%)	χ^2^ = 2.506
Yes	36 (7.5%)	25 (5.1%)	*p* = 0.128	17 (6.3%)	14 (5.8%)	19 (8.3%)	11 (4.8%)	*p* = 0.474
Dream enactment								
No	409 (81.5%)	438 (87.6%)	χ^2^ **=** **7.189**	231 (79.7%)	216 (86.7%)	198 (85.0%)	202 (87.8%)	χ^2^ **=** **8.153**
Yes	93 (18.5%)	62 (12.4%)	**p** **=** **0.007**	59 (20.3%)	33 (13.3%)	35 (15.0%)	28 (12.2%)	**p** **=** **0.043**
Sleep paralysis								
No	417 (83.1%)	435 (87.0%)	χ^2^ = 3.043	234 (80.7%)	209 (83.6%)	198 (85.0%)	212 (92.2%)	χ^2^ **=** **13.927**
Yes	85 (16.9%)	65 (13.0%)	*p* = 0.081	56 (19.3%)	41 (16.4%)	35 (15.0%)	18 (7.8%)	**p** **=** **0.003**
Nightmares								
No	375 (74.7%)	295 (59.1%)	χ^2^ **=** **27.456**	168 (57.9%)	176 (70.7%)	154 (66.4%)	172 (74.8%)	χ^2^ **=** **18.635**
Yes	127 (25.3%)	204 (40.9%)	**p** **<** **0.001**	122 (42.1%)	73 (29.3%)	78 (33.6%)	58 (25.2%)	***p*** **<** **0.001**
Exploding Head Syndrome								
No	450 (89.6%)	443 (88.6%)	χ^2^ = 0.280	251 (86.6%)	214 (85.6%)	209 (90.1%)	219 (95.2%)	χ^2^ **=** **14.212**
Yes	52 (10.4%)	57 (11.4%)	*p* = 0.597	39 (13.4%)	36 (14.4%)	23 (9.9%)	11 (4.8%)	**p** **=** **0.003**

^*^n = 968.

χ^2^, chi-square test.

Statistically significant values (*p* < 0.05) are shown in bold.

**Table 4B:** Current prevalence (at least once during the last 3 months) of different parasomnias in relation to circadian preference and insomnia (*n* = 1,002).

Type of parasomnia	Circadian preference				Insomnia		
	Morning	Intermediate	Evening	Chi-square	No	Yes	Chi-square
Confusional arousals							
No	297 (88.9%)	237 (90.5%)	350 (86.2%)	χ^2^ = 3.005	586 (91.6%)	297 (82.0%)	χ^2^ **=** **20.016**
Yes	37 (11.1%)	25 (9.5%)	56 (13.8%)	*p* = 0.223	54 (8.4%)	65 (18.0%)	**p** **<** **0.001**
Sleepwalking							
No	321 (96.1%)	251 (95.4%)	392 (96.6%)	χ^2^ = 0.530	624 (97.5%)	339 (93.6%)	χ^2^ **=** **9.179**
Yes	13 (3.9%)	12 (4.6%)	14 (3.4%)	*p* = 0.767	16 (2.5%)	23 (6.4%)	**p** **=** **0.002**
Sleep terrors							
No	298 (89.5%)	246 (93.9%)	369 (90.9%)	χ^2^ = 3.635	603 (94.2%)	311 (85.9%)	χ^2^ **=** **19.917**
Yes	35 (10.5%)	16 (6.1%)	37 (9.1%)	*p* = 0.162	37 (5.8%)	51 (14.1%)	**p** **<** **0.001**
Sleep-related eating disorder							
No	330 (99.1%)	256 (97.7%)	402 (99.0%)	χ^2^ = 2.731	636 (99.4%)	353 (97.5%)	χ^2^ **=** **6.255**
Yes	3 (0.9%)	6 (2.3%)	4 (1.0%)	*p* = 0.255	4 (0.6%)	9 (2.5%)	**p** **=** **0.012**
Injured somebody else during sleep							
No	332 (99.4%)	260 (99.2%)	400 (98.5%)	χ^2^ = 1.631	634 (99.1%)	358 (98.9%)	χ^2^ = 0.066
Yes	2 (0.6%)	2 (0.8%)	6 (1.5%)	*p* = 0.442	6 (0.9%)	4 (1.1%)	*p* = 0.798
Injured yourself during sleep							
No	330 (98.8%)	260 (99.2%)	400 (98.3%)	χ^2^ = 0.531	637 (99.5%)	351 (97.2%)	χ^2^ **=** **9.536**
Yes	4 (1.2%)	3 (1.1%)	7 (1.7%)	*p* = 0.767	3 (0.5%)	10 (2.8%)	**p** **=** **0.002**
Sexsomnia^*^							
No	292 (91.0%)	242 (97.2%)	373 (93.7%)	χ^2^ **=** **9.197**	579 (93.5%)	329 (94.0%)	χ^2^ = 0.081
Yes	29 (9.0%)	7 (2.8%)	25 (6.3%)	**p** **=** **0.010**	40 (6.5%)	21 (6.0%)	*p* = 0.776
Dream enactment							
No	283 (85.0%)	222 (84.7%)	342 (84.2%)	χ^2^ = 0.139	547 (85.5%)	300 (82.9%)	χ^2^ = 1.191
Yes	50 (15.0%)	40 (15.3%)	65 (16.0%)	*p* = 0.933	93 (14.5%)	62 (17.1%)	*p* = 0.275
Sleep paralysis							
No	287 (86.2%)	222 (84.7%)	342 (84.2%)	χ^2^ = 0.568	574 (89.7%)	278 (76.8%)	χ^2^ **=** **30.190**
Yes	46 (13.8%)	40 (15.3%)	64 (15.8%)	*p* = 0.753	66 (10.3%)	84 (23.2%)	**p** **<** **0.001**
Nightmares							
No	224 (67.1%)	179 (68.3%)	267 (65.8%)	χ^2^ = 0.479	478 (74.7%)	193 (53.3%)	χ^2^ **=47.744**
Yes	110 (32.9%)	83 (31.7%)	139 (34.2%)	*p* = 0.787	162 (25.3%)	169 (46.7%)	**p** **<** **0.001**
Exploding head syndrome							
No	304 (91.3%)	241 (92.0%)	348 (85.5%)	χ^2^ **=** **9.327**	600 (93.8%)	293 (80.9%)	χ^2^ **=** **39.141**
Yes	29 (8.7%)	21 (8.0%)	59 (14.5%)	**p** **=** **0.009**	40 (6.3%)	69 (19.1%)	**p** **<** **0.001**

^*^n = 968.

χ^2^, chi-square test.

Statistically significant values (*p* < 0.05) are shown in bold.

**Table 4C:** Current prevalence (at least once during the last 3 months) of different parasomnias in relation to anxiety and depression (*n* = 1,002).

Type of parasomnia	Anxiety			Depression		
	No	Yes	Chi-square	No	Yes	Chi-square
Confusional arousals						
No	768 (89.9%)	115 (77.7%)	χ^2^ **=** **18.019**	747 (89.6%)	137 (81.1%)	χ^2^ **=** **9.717**
Yes	86 (10.1%)	33 (22.3%)	**p** **<** **0.001**	87 (10.4%)	32 (18.9%)	**p** **=** **0.002**
Sleepwalking						
No	829 (97.1%)	135 (91.2%)	χ^2^ **=** **11.857**	809 (97.1%)	154 (91.7%)	χ^2^ **=** **11.379**
Yes	25 (2.9%)	13 (8.8%)	***p*** **<** **0.001**	24 (2.9%)	14 (8.3%)	***p*** **<** **0.001**
Sleep terrors						
No	790 (92.5%)	124 (83.8%)	χ^2^ **=** **11.978**	770 (92.4%)	143 (84.6%)	χ^2^ **=** **10.620**
Yes	64 (7.5%)	24 (16.2%)	**p** **<** **0.001**	63 (7.6%)	26 (15.4%)	**p** **=** **0.001**
Sleep-related eating disorder						
No	842 (98.6%)	147 (99.3%)	χ^2^ = 0.524	822 (98.7%)	167 (98.8%)	χ^2^ = 0.021
Yes	12 (1.4%)	1 (0.7%)	*p* = 0.469	11 (1.3%)	2 (1.2%)	*p* = 0.886
Injured somebody else during sleep						
No	847 (99.2%)	145 (98.0%)	χ^2^ = 1.861	825 (99.0%)	167 (99.4%)	χ^2^ = 0.209
Yes	7 (0.8%)	3 (2.0%)	*p* = 0.173	8 (1.0%)	1 (0.6%)	*p* = 0.647
Injured yourself during sleep						
No	845 (98.9%)	144 (97.3%)	χ^2^ = 2.678	827 (99.3%)	162 (95.9%)	χ^2^ **=** **12.845**
Yes	9 (1.1%)	4 (2.7%)	*p* = 0.102	6 (0.7%)	7 (4.1%)	**p** **<** **0.001**
Sexsomnia^*^						
No	776 (93.8%)	132 (93.0%)	χ^2^ = 0.157	756 (93.9%)	151 (92.6%)	χ^2^ = 0.373
Yes	51 (6.2%)	10 (7.0%)	*p* = 0.692	49 (6.1%)	12 (7.4%)	*p* = 0.541
Dream enactment						
No	725 (84.9%)	122 (82.4%)	χ^2^ = 0.585	710 (85.2%)	137 (81.1%)	χ^2^ = 1.867
Yes	129 (15.1%)	26 (17.6%)	*p* = 0.444	123 (14.8%)	32 (18.9%)	*p* = 0.172
Sleep paralysis						
No	743 (87.0%)	108 (73.0%)	χ^2^ **=** **19.398**	723 (86.7%)	129 (76.8%)	χ^2^ **=** **10.777**
Yes	111 (13.0%)	40 (27.0%)	***p*** **<** **0.001**	111 (13.3%)	39 (23.2%)	***p*** **=** **0.001**
Nightmares						
No	612 (71.7%)	59 (39.9%)	χ^2^ **=** **57.655**	600 (71.9%)	71 (42.0%)	χ^2^ **=** **56.849**
Yes	242 (28.3%)	89 (60.1%)	***p*** **<** **0.001**	234 (28.1%)	98 (58.0%)	***p*** **<** **0.001**
Exploding head syndrome						
No	780 (91.3%)	113 (76.4%)	χ^2^ **=** **29.210**	760 (91.1%)	134 (79.3%)	χ^2^ **=** **20.328**
Yes	74 (8.7%)	35 (23.6%)	***p*** **<** **0.001**	74 (8.9%)	35 (20.7%)	***p*** **<** **0.001**

^*^n = 968.

χ^2^, chi-square test.

Statistically significant values (*p* < 0.05) are shown in bold.

The current prevalence of most parasomnias was notably higher among individuals with insomnia. Confusional arousals, sleepwalking, sleep terrors, sleep paralysis, and exploding head syndrome were two to three times more common in this group, while sleep-related eating disorder and self-injury were four to five times more prevalent among those with insomnia (Table 4). Nightmares were almost twice as common in participants with insomnia compared to those without.

Participants with anxiety showed a two to three times higher prevalence of confusional arousals, sleepwalking, sleep terrors, sleep paralysis, nightmares, and exploding head syndrome than those without anxiety (Table 4). Among individuals with depression, the prevalence of sleepwalking, sleep terrors, nightmares, and exploding head syndrome was also two to three times higher than among those without depression. Self-injury was almost six times more common in this group, while confusional arousals and sleep paralysis were almost twice as prevalent among the group with depression compared with the group without.

In the adjusted logistic regression analyses, the total insomnia score was a significant predictor for current confusional arousals, sleepwalking, sleep terrors, sleep-related eating disorder, self-injury, sleep paralysis, nightmares, and exploding head syndrome (Table 5). Sleep-related eating disorder showed the strongest association with the insomnia score (OR = 1.08), indicating an 8% increase in odds per point increase on the insomnia scale. The composite total anxiety score was a significant predictor for current confusional arousals, sleepwalking, sleep terrors, self-injury, dream enactment, sleep paralysis, nightmares, and exploding head syndrome (Table 5). Nightmares had the strongest association with the anxiety score. The odds of experiencing repeated and terrible nightmares increased by 58% for every point scored on the anxiety scale. The depression score was a significant predictor for current confusional arousals, sleep terrors, sleep paralysis, nightmares, and exploding head syndrome (Table 5), in the adjusted logistic regression analyses. Nightmares had the strongest association with the depression score. The odds of experiencing repeated and terrible nightmares increased by 44% for every point scored on the depression scale.

**Table 5:** Current prevalence (at least once during the last 3 months) of different parasomnias in relation to sociodemographic variables, circadian preference, total insomnia score, total anxiety score and total depression score (*n* = 1,002).

Characteristic	Confusional arousals OR (95% CI)		Sleepwalking OR (95% CI)		Sleep terrors OR (95% CI)		Sleep-related eating disorder OR (95% CI)	
	Crude	Adjusted	Crude	Adjusted	Crude	Adjusted	Crude	Adjusted
Sex								
Male	1.0 (ref)		1.0 (ref)		1.0 (ref)		1.0 (ref)	
Female	0.74 (0.51–1.09)		0.58 (0.30–1.13)		0.89 (0.57–1.37)		1.06 (0.36–3.15)	
Age (continuous variable)	**0.98 (0.97**–**0.99)**		**0.98 (0.96**–**1.00)**		1.00 (0.99–1.01)		1.01 (0.98–1.04)	
Education^**^								
Low	1.0 (ref)		1.0 (ref)		1.0 (ref)		1.0 (ref)	
Intermediate	1.30 (0.76–2.20)		0.58 (0.24–1.42)		0.88 (0.49–1.56)		1.27 (0.34–4.76)	
High	0.66 (0.42–1.06)		**0.39 (0.19**–**0.80)**		**0.42 (0.25–0.70)**		0.40 (0.10–1.52)	
Circadian preference								
Morning type	1.0 (ref)		1.0 (ref)		1.0 (ref)		1.0 (ref)	
Intermediate type	0.86 (0.50–1.46)		1.16 (0.51–2.62)		0.55 (0.30–1.01)		2.28 (0.59–8.81)	
Evening type	1.29 (0.83–2.01)		0.93 (0.43–2.01)		0.85 (0.52–1.38)		0.95 (0.22–4.12)	
Bergen Insomnia Scale	**1.05 (1.03**–**1.07)**	**1.05 (1.03**–**1.07)**	**1.04 (1.01**–**1.07)**	**1.04 (1.01**–**1.07)**	**1.05 (1.03**–**1.07)**	**1.05 (1.03**–**1.08)**	**1.08 (1.03**–**1.14)**	**1.08 (1.03**–**1.14)**
Anxiety score	**1.26 (1.12**–**1.40)**	**1.22 (1.09**–**1.38)**	**1.28 (1.07**–**1.53)**	**1.27 (1.05**–**1.53)**	**1.27 (1.12**–**1.43)**	**1.31 (1.15**–**1.50)**	1.04 (0.74–1.48)	1.04 (0.73–1.50)
Depression score	**1.26 (1.12**–**1.41)**	**1.21 (1.07**–**1.36)**	**1.25 (1.04**–**1.50)**	1.21 (0.99–1.46)	**1.26 (1.11**–**1.43)**	**1.30 (1.13**–**1.49)**	1.11 (0.79–1.54)	1.11 (0.78–1.58)
**Characteristic**	**Injured somebody else during sleep OR (95% CI)**		**Injured yourself during sleep OR (95% CI)**		**Sexsomnia OR (95% CI)** ^*^		**Dream enactment OR (95% CI)**	
	**Crude**	**Adjusted**	**Crude**	**Adjusted**	**Crude**	**Adjusted**	**Crude**	**Adjusted**
Sex								
Male	1.0 (ref)		1.0 (ref)		1.0 (ref)		1.0 (ref)	
Female	2.80 (0.67–11.65)		1.92 (0.61–6.04)		0.68 (0.40–1.15)		**0.62 (0.44**–**0.88)**	
Age (continuous variable)	0.97 (0.94–1.01)		**0.95 (0.91**–**0.99)**		1.00 (0.99–1.01)		**0.99 (0.98**–**1.00)**	
Education^**^								
Low	1.0 (ref)		1.0 (ref)		1.0 (ref)		1.0 (ref)	
Intermediate	1.32 (0.23–7.54)		0.81 (0.16–4.06)		1.45 (0.70–2.99)		0.94 (0.58–1.52)	
High	0.79 (0.17–3.69)		0.75 (0.21–2.63)		0.80 (0.42–1.53)		**0.59 (0.39**–**0.88)**	
Morning type	1.0 (ref)		1.0 (ref)		1.0 (ref)		1.0 (ref)	
Intermediate type	1.50 (0.20–11.31)		0.84 (0.17–4.12)		**0.28 (0.12**–**0.66)**		1.03 (0.66–1.61)	
Evening type	2.79 (0.51–15.12)		1.42 (0.40–5.00)		0.68 (0.39–1.18)		1.07 (0.71–1.59)	
Bergen Insomnia Scale	0.99 (0.93–1.06)	0.96 (0.89–1.04)	**1.08 (1.03**–**1.13)**	**1.07 (1.02**–**1.13)**	1.00 (0.98–1.03)	1.01 (0.98–1.03)	1.01 (1.00–1.03)	1.01 (1.00–1.03)
Anxiety score	1.19 (0.83–1.70)	1.04 (0.71–1.51)	**1.52 (1.16**–**1.98)**	**1.37 (1.02**–**1.84)**	1.03 (0.87–1.22)	1.06 (0.89–1.27)	**1.14 (1.02**–**1.26)**	**1.13 (1.01**–**1.27)**
Depression score	1.01 (0.67–1.53)	0.89 (0.58–1.36)	**1.48 (1.12**–**1.95)**	1.34 (0.99–1.80)	1.02 (0.86–1.21)	1.05 (0.87–1.26)	1.04 (0.93–1.16)	1.02 (0.90–1.14)
**Characteristic**	**Sleep paralysis OR (95% CI)**		**Nightmares OR (95% CI)**		**Exploding Head Syndrome OR (95% CI)**		
	Crude	Adjusted	Crude	Adjusted	Crude		Adjusted	
Sex								
Male	1.0 (ref)		1.0 (ref)		1.0 (ref)			
Female	0.73 (0.51–1.03)		**2.05 (1.56**–**2.68)**		1.10 (0.74–1.64)			
Age (continuous variable)	**0.98 (0.97**–**0.99)**		**0.99 (0.98**–**0.99)**		**0.98 (0.97**–**0.99)**			
Education^**^								
Low	1.0 (ref)		1.0 (ref)		1.0 (ref)			
Intermediate	0.81 (0.50–1.32)		0.97 (0.66–1.44)		1.42 (0.80–2.51)			
High	**0.51 (0.34**–**0.76)**		0.74 (0.54–1.01)		0.86 (0.52–1.40)			
Circadian preference								
Morning type	1.0 (ref)		1.0 (ref)		1.0 (ref)			
Intermediate type	1.11 (0.70–1.76)		0.94 (0.67–1.34)		0.91 (0.50–1.63)			
Evening type	1.16 (0.77–1.75)		1.06 (0.78–1.44)		**1.77 (1.11**–**2.84)**			
Bergen Insomnia Scale	**1.05 (1.03**–**1.07)**	**1.05 (1.03**–**1.07)**	**1.06 (1.05–1.08)**	**1.05 (1.04**–**1.07)**	**1.07 (1.05**–**1.09)**		**1.07 (1.05**–**1.09)**	
Anxiety score	**1.32 (1.19**–**1.46)**	**1.30 (1.17**–**1.46)**	**1.64 (1.48**–**1.81)**	**1.58 (1.42**–**1.74)**	**1.44 (1.29**–**1.61)**		**1.42 (1.26**–**1.60)**	
Depression score	**1.32 (1.19**–**1.47)**	**1.29 (1.16**–**1.44)**	**1.49 (1.35**–**1.63)**	**1.44 (1.31**–**1.59)**	**1.46 (1.30**–**1.63)**		**1.41 (1.25**–**1.60)**	

^*^n = 968.

^**^n = 995.

OR, odds ratio; CI, confidence interval.

Statistically significant values (*p* < 0.05) are shown in bold.

Sex, age, educational level, and circadian preference were included as covariates in each of the adjusted analyses.

## 4 Discussion

This cross-sectional epidemiological survey provides new insight into the prevalence of different parasomnias in the general Norwegian adult population, as well as the associations between parasomnias and demographic factors and mental health conditions. Current prevalence varied from 1.0% (injured somebody else during sleep) to 33.1% (nightmares). Not surprisingly, the data showed a clear association between several of the parasomnias and insomnia, anxiety, and depression.

The lifetime prevalence of the different parasomnias was comparable to those found 16 years ago (Bjorvatn et al., 2010). The most notable difference between the studies was the prevalence of nightmares. In our study, 47.2% of the participants had experienced this parasomnia, which was lower than the 66.2% in the previous study. The different phrasing of the question regarding nightmares, which this time defined it as repeated and terrible nightmares, in accordance with the criteria of nightmare disorder, likely explains this discrepancy. In general, the current prevalence of the different parasomnias was higher than in the previous study (Bjorvatn et al., 2010), which may be explained by the high rates of depression and insomnia in the present study (Alshahrani et al., 2023; Hanif et al., 2024).

Sleep paralysis showed a lifetime prevalence of 18.8%, notably higher than the 7.6% reported in a previous systematic review (Sharpless and Barber, 2011). However, a more recent study found 1-year prevalence rates of 9.7% and 15.1% (Ohayon and Pakpour, 2022), indicating that the occurrence of this parasomnia may be more common than earlier estimates suggest. Nevertheless, further research is needed to elucidate the true prevalence of sleep paralysis. In the case of exploding head syndrome, the lifetime prevalence was 12.9%, which is comparable to 10.8% found in a study from Germany (Fulda et al., 2008). However, other studies have shown rates of 29.6% and 52.7% (Denis et al., 2019; Sharpless et al., 2020). These discrepancies underscore the uncertainty surrounding the true prevalence of this parasomnia, likely due to limited research, differences in assessment methods, and low clinical awareness.

Several studies have found that the prevalence of NREM parasomnias and nightmares tends to decrease with older age (Nielsen et al., 2006; Ohayon et al., 1999; Ohayon and Schenck, 2010; Takaesu et al., 2016; Trajanovic et al., 2007). In our study, we replicated this pattern for confusional arousals, sleepwalking, and nightmares, while sleep terrors, sleep-related eating disorder, and sexsomnia showed no age-related differences. We also found an association between younger age and self-injury during sleep, but not with injuring others, thus only partly reproducing earlier findings on violent behavior during sleep. Furthermore, sleep paralysis and exploding head syndrome were more common among younger respondents, contrary to earlier literature (Denis et al., 2019; Sharpless and Barber, 2011). Surprisingly, we found a clear association between younger age and dream enactment. This could reflect that dream-related processes also occur during NREM parasomnias (Castelnovo et al., 2024; Idir et al., 2022), and/or possible underreporting by older individuals. When exploring the association between age and lifetime prevalence of the different parasomnias, we found many of the same results as for current prevalence. These results seem counterintuitive, as you would expect older participants to have experienced more parasomnias during their lifetime than younger. This could reflect recall bias among the older participants. Therefore, we advise caution when interpreting the association between age and lifetime prevalence of parasomnias in the present study. Underreporting among elderly would conceivably also lead to an underestimation of the general prevalence of parasomnias.

In our study, women reported nightmares more frequently than men, which aligns with previous findings (Bjorvatn et al., 2010; Schredl and Reinhard, 2011). Increased neuroticism in female participants has been proposed as a mediating factor for these findings, but this is likely not the whole story (Schredl, 2014; Schredl and Goeritz, 2019). Another parasomnia showing a sex difference in our study was dream enactment, which was more commonly reported by men. Dream enactment is a hallmark feature of REM sleep behavior disorder (RBD), which has been found to be associated with male sex in a recent systematic review (Schredl and Reinhard, 2011). However, while several previous studies relied on polysomnographic verification, our assessment was based on a single self-report item. Thus, our measure likely captures a broader spectrum of dream enactment behaviors, including those related to NREM parasomnias (Castelnovo et al., 2024; Idir et al., 2022). As such, not all cases in our sample may reflect true RBD. Additionally, underreporting by older participants may have limited the true number of participants reporting RBD-associated dream enactment, a possibility discussed above.

Somewhat surprisingly, the results did not show a clear association between circadian preference and parasomnias. This finding contrasts with a previous study suggesting that evening chronotype may be associated with certain parasomnia-related phenomena, such as greater nightmare distress (Choo et al., 2023). Nonetheless, parasomnias such as exploding head syndrome have been examined in relation to chronotype, with no significant association being found (Kirwan and Fortune, 2021). Chronobiological mechanisms have been proposed to contribute to NREM-parasomnias due to their sensitivity to sleep timing (Mainieri et al., 2021). Evening preference may cause curtailed sleep, especially during the weekdays (Minz and Pati, 2021), which is a known risk factor for NREM-related parasomnias (Pressman, 2007). The lack of significant associations in the present study may reflect limited statistical power or differences in study populations and measurement approaches.

There are a multitude of studies showing an association between insomnia and parasomnias such as confusional arousals (Ohayon et al., 1999, 2000), sleepwalking (Lopez et al., 2013; Ohayon et al., 2012), sleep terrors (Ohayon et al., 1999), RBD (Arnulf et al., 2015), sleep paralysis (Denis et al., 2018), nightmares (Li et al., 2010; Ollila et al., 2024; Sandman et al., 2015) and exploding head syndrome (Denis et al., 2019; Sharpless et al., 2020; Faccini and Del-Monte, 2024). Our findings support these results, as insomnia symptoms were significantly associated with most of the parasomnias, with the exception of injury to others during sleep, sexsomnia, and dream enactment. Although the ORs may appear modest at first glance, they capture the effect across the entire 42-point insomnia scale, and should therefore be interpreted as clinically and statistically meaningful. This broad association contrasts with findings from our 2010 study, where only confusional arousals and sleep terrors were linked to insomnia (Bjorvatn et al., 2010). However, the 2010 article used lifetime prevalence for the analyses, whereas the present study focused on parasomnias reported during the last 3 months. The strong overall association between insomnia and parasomnias raises important questions about how they intercorrelate. Because these parasomnias typically involve partial arousals or awakenings from sleep, they may directly fragment sleep and thereby exacerbate insomnia symptoms. Insomnia is also believed to prime sleepwalking in participants susceptible to this disorder (Pressman, 2007). In addition, many people with insomnia use sleep medications, some of which, such as zolpidem, have been shown to trigger parasomnias (Ho et al., 2020; Mittal et al., 2021; Stallman et al., 2018). Future research should explore not only the co-occurrence of insomnia and parasomnias, but the causal pathways linking them.

Depression and anxiety were significantly associated with several parasomnias. The only parasomnias not significantly associated with anxiety were sleep-related eating disorder, injury to others during sleep, and sexsomnia. Depression was significantly associated with the same parasomnias as anxiety, with the exceptions of sleepwalking, self-injury, and dream enactment. Notably, the same parasomnias that were associated with depression in the 2010 study, also had significant associations in the present study, except for injuring oneself during sleep (Bjorvatn et al., 2010). Previous studies have also reported associations between anxiety and depression and NREM-related parasomnias. In line with this literature, we replicated these findings for confusional arousals and sleep terrors (Ohayon et al., 1999, 2000). Although sleepwalking has previously been associated with major depressive disorder in a large U.S. sample (Ohayon et al., 2012), we did not observe an association with depression, but rather with anxiety. Similarly a large-scale study on violent behavior during sleep found an association with mood disorders, but not anxiety (Ohayon and Schenck, 2010), whereas in our study, injuring oneself during sleep was linked to anxiety, and no associations were found for injuring others. We were also unable to replicate previous studies that show an association between mental disorders and sleep-related eating disorder (Hanif et al., 2024; Winkelman et al., 1999) and sexsomnia (Trajanovic et al., 2007; Alejandra and Alejandro, 2019). Similarly to the NREM-related parasomnias, the REM-related parasomnias have previously been linked to depression in meta-analyses and systematic reviews (Denis et al., 2018; Faccini and Del-Monte, 2024; Wu et al., 2022). These studies also reported similar associations with anxiety, although not for RBD. While we replicated the associations for nightmares and sleep paralysis, we did not replicate the findings for dream enactment, likely due to the methodological issues previously mentioned. Given that depression is known to alter REM sleep architecture (Crişan et al., 2024; Palagini et al., 2013), this may offer a plausible explanation for its association with REM-related parasomnias, though the underlying mechanisms warrant further investigation. Lastly, we found that exploding head syndrome was strongly associated with both mental disorders, but the available literature is more mixed regarding these associations (Denis et al., 2019; Kirwan and Fortune, 2021). Overall, our findings support a strong association between mental disorders and parasomnias. Recognizing these associations may help clinicians identify underlying psychiatric symptoms in patients presenting with parasomnia symptoms, and conversely, increase awareness of parasomnias in individuals with anxiety or depression, supporting the need for integrated assessment and care.

Our study has several strengths and limitations that deserve mention. First, a large sample (*n* = 1,002) was acquired through a web-panel, believed to reflect the general Norwegian adult population in terms of sex, age, and geography. The data were weighted to adjust for potential discrepancies between the sample and the true population. Second, we used validated questionnaires to assess the symptoms of insomnia, depression, and anxiety (Pallesen et al., 2008; Löwe et al., 2010). However, only one, non-validated item was used for assessing circadian preference. Nonetheless, this single circadian morningness/eveningness item is often used in epidemiological research targeting larger sample sizes (Merikanto et al., 2022). For the chi-square tests, the scores were categorized into relevant groups. Furthermore, in the regression analyses, the full continuous scale scores for insomnia, anxiety, and depression were used to reduce information loss. Regarding the measurement of insomnia symptoms, it should be acknowledged that no clinical interviews were performed, making it possible that other sleep disorders were the source of the symptoms. The same applies for the anxiety and depression symptoms to other disorders, as they were only measured through PHQ-4. It should also be mentioned that there were no questions regarding medication use. This is especially limiting when interpreting the association between insomnia and parasomnias, since hypnotic medications such as zolpidem are known to be associated with parasomnias. There were also no questions regarding alcohol use or relevant medical history. Since both increased alcohol intake and neurological conditions like Parkinson's disease are associated with poor mental health (Puddephatt et al., 2022; Aarsland et al., 2009), insomnia (Husberg et al., 2022; Duan et al., 2025) and certain parasomnias (Ohayon et al., 1999; St Louis et al., 2017; Ohayon et al., 2012), this could represent a hidden confounder in our data. Furthermore, no information regarding other sleep disorders was included. Certain disorders, such as obstructive sleep apnea (OSA), may trigger nocturnal arousals and parasomnia-related behavior and may therefore represent potential confounders (Mainieri et al., 2021; Pressman, 2007; Kimoff, 1996; Iranzo and Santamaría, 2005). However, a Norwegian study from 2018 did not find any increased prevalence of parasomnias among patients with OSA compared with individuals without OSA (Lundetræ et al., 2018). Nonetheless, the absence of information on other sleep disorders should be considered when interpreting the current findings. Another major limitation was the low response rate, warranting caution when interpreting the results. Furthermore, there is a possibility of selection bias tied to the invitation sent out before taking the survey, as potential respondents were informed that the survey included questions regarding sleep phenomena and sleep habits. The chance of our sample having a higher prevalence of sleep-related disturbances because of the information given beforehand, must be taken into consideration. Still, we believe that the reported associations between parasomnias and insomnia, anxiety, and depression are valid. Furthermore, we recognize that all data are based on self-report. Recall bias could have had an impact, especially when gathering information regarding lifetime prevalence. This fact, in combination with the relatively simple and non-specific phrasing of the parasomnia questions, increases the risk for potential inconsistencies. Whereas the ICSD-3 and DSM-5 define different parasomnias through multiple sets of symptoms, our data were based on the main symptoms of each parasomnia. Accordingly, we acknowledge the risk that some respondents may have misinterpreted the phrasing of some of the questions, and therefore potentially skewed the data. As all data was collected at one time point and with the one method (self-report), the observed associations between parasomnias and the other variables may additionally reflect common method bias (Podsakoff et al., 2024). There is also a risk of results being significant by chance, since tests were conducted on many outcomes. Because the present work was designed as an exploratory analysis, we did not apply formal statistical corrections for multiple testing. Lastly, when comparing the groups with and without current parasomnias, there were few participants in some groups, limiting the statistical power.

To conclude, this cross-sectional epidemiological survey provides new insight into the prevalence of different parasomnias and their correlates in the Norwegian adult population. Several parasomnias were highly prevalent both in terms of lifetime and current occurrence. Notably, strong associations were observed between multiple parasomnias and symptoms of insomnia, anxiety, and depression. These findings could have major implications for understanding how parasomnias originate, and how they are maintained, as well as what co-morbidities commonly occur. Henceforth it could suggest a multi-disciplinary approach to the diagnosing and treatment of parasomnias, as well as a further emphasis on recognizing parasomnias in vulnerable patient groups. Further research is needed to explore the interplay between the variables and their association with parasomnias.

## Data Availability

The raw data supporting the conclusions of this article will be made available by the authors, without undue reservation.
